# Dataset for genome sequencing and de novo assembly of the Vietnamese bighead catfish (*Clarias macrocephalus* Günther, 1864)

**DOI:** 10.1016/j.dib.2020.105861

**Published:** 2020-06-16

**Authors:** Thuy-Yen Duong, Mun Hua Tan, Yin Peng Lee, Larry Croft, Christopher M. Austin

**Affiliations:** a College of Aquaculture and Fisheries, Can Tho University, Can Tho City, Viet Nam; b Deakin Genomics Centre, Deakin University, Geelong 3220, Victoria, Australia; c Centre for Integrative Ecology, School of Life and Environmental Sciences, Deakin University, Geelong 3220, Victoria, Australia

**Keywords:** *Clarias macrocephalus*, Genome, Illumina, Catfish, Aquaculture

## Abstract

Freshwater catfish of the genus *Clarias*, known as the airbreathing catfish, are widespread and important for food security through small scale inland fisheries and aquaculture. Limited genomic data are available for this important group of fishes. The bighead catfish (*Clarias macrocephalus*) is a commercial aquaculture species in southeast Asia used for aquaculture and threatened in its natural environment through habitat destruction, over-exploitation and competition from other introduced species of *Clarias*. Despite its commercial importance and threats to natural populations, public databases do not include any genomic data for *C. macrocephalus*. We present the first genomic data for the bighead catfish from Illumina sequencing. A total of 128 Gb of sequence data in paired-end 150 bp reads were assembled *de novo*, generating a final assembly of 883 Mbp contained in 27,833 scaffolds (N_50_ length: 80.8 kbp) with BUSCO completeness assessments of 96.3% and 87.6% based on metazoan and Actinopterygii ortholog datasets, respectively. Annotation of the genome predicted 21,124 gene sequences, which were assigned putative functions based on homology to existing protein sequences in public databases. Raw fastq reads and the final version of the genome assembly have been deposited in the NCBI (BioProject: PRJNA604477, WGS: JAAGKR000000000, SRA: SRR11188453). The complete *C. macrocephalus* mitochondrial genome was also recovered from the same sequence read dataset and is available on NCBI (accession: MT109097), representing the first mitogenome for this species. Lastly, we find an expansion of the *mb* and *ora1* genes thought to be associated with adaptations to air-breathing and a semi-terrestrial life style in this genus of catfish.

**Specifications Table**

**Table:** 

**Subject**	Biology
**Specific subject area**	Genomics
**Type of data**	Sequencing raw reads, Assembly, Table, Figure,
**How data were acquired**	Illumina NovaSeq
**Data format**	Raw Reads (fastq), Assembly (fasta), Protein and Transcript sequences (fasta)
**Parameters for data collection**	DNA from a white muscle tissue sample of an adult catfish specimen was used for library preparation and sequencing.
**Description of data collection**	Total genomic DNA extraction was performed using the SDS-Chloroform extraction method. The gDNA library was subsequently processed with the Illumina TruSeq PCR Free kit following manufacturer's instructions. Paired-end sequencing of the constructed library was performed on a NovaSeq 6000 (2 × 150 bp run configuration) at the Deakin Genomics Centre.
**Data source location**	9° 19′ 28.9″ N; 104° 53′ 20.9″ E
**Data accessibility**	Mitochondrial genome is available on NCBI under accession number MT109097 (https://www.ncbi.nlm.nih.gov/nuccore/MT109097). Raw data and final assembled contigs were deposited in the NCBI database under BioProject: PRJNA604477 (https://www.ncbi.nlm.nih.gov/bioproject/PRJNA604477), WGS: JAAGKR000000000 (https://www.ncbi.nlm.nih.gov/nuccore/1821738013), SRA: SRR11188453 (https://www.ncbi.nlm.nih.gov/sra?linkname=bioproject_sra_all&from_uid=604477). Additional files including BUSCO analysis output, genome repeat profile, predicted protein and transcript sequences are available in a public repository (doi: 10.17632/d8739nckf9.2).

**Value of the Data**

First genomic dataset for the wild bighead catfish
High BUSCO completeness and an assembly close to the estimated genome size indicate it will enable its use in selective breeding and population and conservation genetic studies of this native Vietnamese and commercial species.
The data will facilitate genetic management for the genetic improvement and the conservation of bighead catfish populations including competition with introduced non-native species of *Clarias*.
The data adds to the limited genomic available for the highly diverse catfish lineage [1], [2], [3], [4]

## 1 Data description

**Fig. 1:**
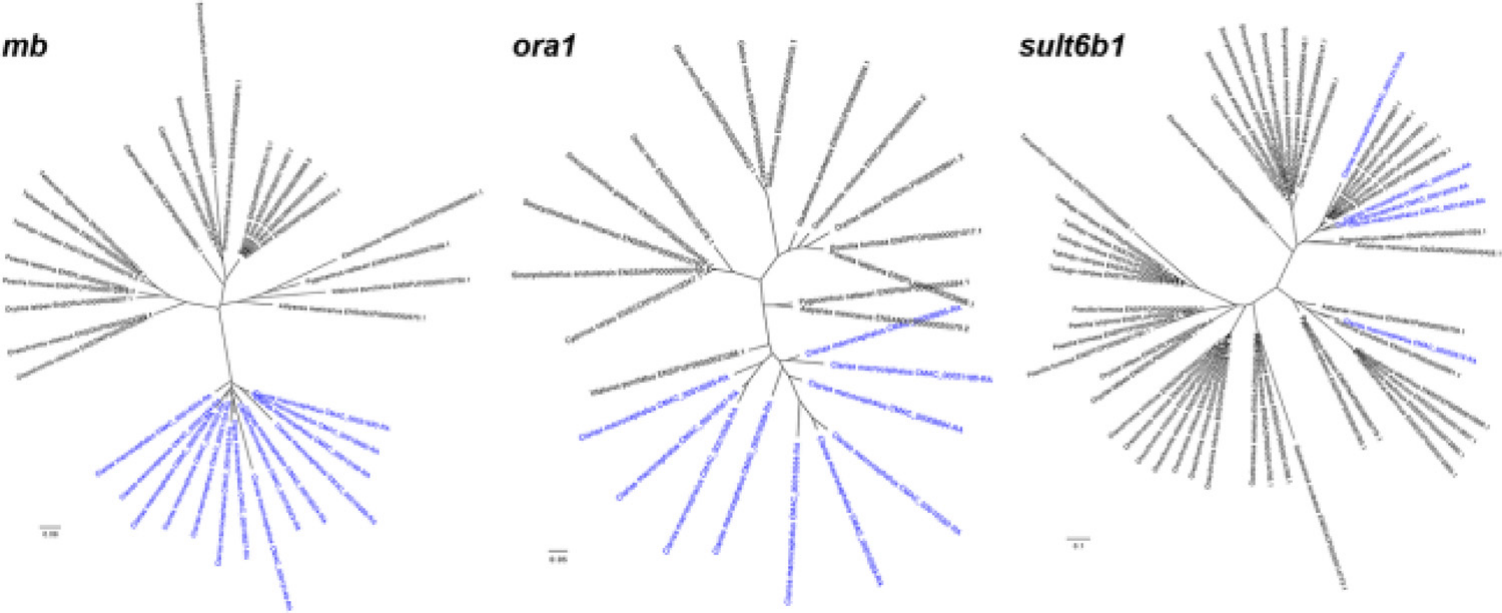
Gene copies of *mb, ora1* and *sult6b1* in genomes of *Clarias macrocephalus* and other fish (*C. macrocephalus* genes colored in blue).

**Fig. 2:**
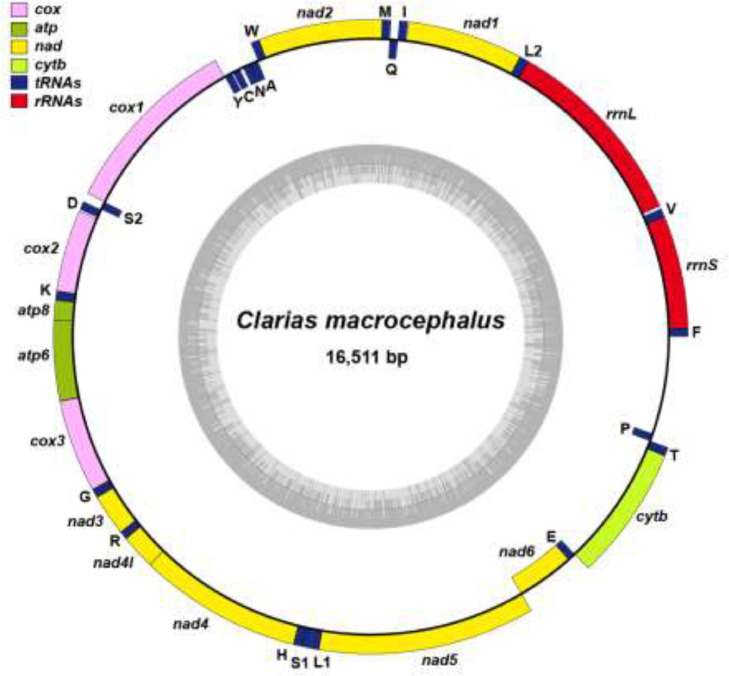
Mitochondrial genome of *Clarias macrocephalus*.

DNA sequencing using the Illumina platform was performed to generate the first *de novo* genome assembly for the commercial bighead catfish (*Clarias macrocephalus*). Approximately 853 million paired-end 150-bp reads were generated (128 GB of total bases) on a NovaSeq at the Deakin Genomics Centre. After poly-G, adapter and quality trimming, 98.75% of reads were retained and subsequently used for *de novo* assembly. The final 883 Mbp assembly consists of 27,833 scaffolds and contains 96.3% and 87.6% of complete metazoan and Actinopterygii BUSCOs, respectively (Table 1). A kmer-based method estimates a heterozygosity rate of 0.5% and approximately 70.7 to 81% unique content in the genome. Subsequently, a total of 21,124 gene sequences were predicted from the genome, based on alignment evidence from protein sequences in SwissProt and *C. macrocephalus* transcript sequences assembled from short reads available on NCBI's SRA database (Table 1). Significant expansions of each of the *mb* and *ora1* genes were observed (Fig. 1). The mitochondrial genome was extracted from the data set, which is 16,511 bp and is fully annotated with no gaps and contains 37 genes (Fig. 2).

**Table 1:** Sequencing, assembly and annotation of the *Clarias macrocephalus* genome.

**Estimated genome size**	
Based on 19-mers	883,670,904 bp
Based on 21-mers	880,824,886 bp
Based on 25-mers	878,508,361 bp
***De novo* assembly (MaSuRCA + purge_haplotigs)**	
Number of scaffolds	27,833
Assembly size	883,399,353 bp
Average scaffold size	31,739.28 bp
Scaffold N_50_ size	80,802 bp
Largest scaffold	650,799 bp
Smallest scaffold	200 bp
Number of Ns	998,952 bp
Number of gaps	17,036
Percentage of short reads aligned to assembly	96.14%
**BUSCO (metazoa, *n*** **=** **978)**	
Complete	942 (96.3%)
Complete and single copy	900 (92.0%)
Complete and duplicated copy	42 (4.3%)
Fragmented	21 (2.1%)
Missing	15 (1.6%)
**BUSCO (actinopterygii, *n*** **=** **4584)**	
Complete	4014 (87.6%)
Complete and single copy	3859 (84.2%)
Complete and duplicated copy	155 (3.4%)
Fragmented	294 (6.4%)
Missing	276 (6.0%)
**Annotation**	
Number of predicted genes (AED ≤ 0.5)	21,124
Number of genes with homology to NR	20,693 (98.0%)
Number of genes with functional domain	20,794 (98.4%)

## 2 Experimental design, materials, and methods

### 2.1 Catfish, tissue sampling and DNA extraction

A white muscle tissue sample was obtained from an adult catfish collected from a natural population which was used for aquaculture trials at Can Tho University, Viet Nam. The fish was anaesthetized in AQUI-S (20 ppm) before sampling. The tissue was preserved in ethanol and approximately 50 mg was used for genomic DNA (gDNA) extractions using a modified SDS-chloroform method (Sokolov, 2000). In brief, the tissue sample was cut into smaller pieces before an overnight digestion in 380µL lysis buffer (50 mM Tris–HCl, 10 mM EDTA, 20% SDS) and 20 µL Proteinase K (>600 mAU/mL). Protein precipitation was performed by adding 100 µL saturated >5 M KCl and incubated on ice. 1x volume of chloroform was added and the aqueous layer containing the DNA was transferred into a new tube. DNA was precipitated using 1x volume of isopropanol and washed using 1 mL 80% ethanol before being eluted in 100 µL of elution buffer (10 mM Tris–HCl, 1 mM EDTA).

### 2.2 DNA library construction and sequencing

Approximately 2 μg of total DNA as measured by the Qubit 3.0 fluorometer (Invitrogen, USA) was used as the input for TruSeq PCR-Free kit (Illumina, San Diego, CA). Library was quantified using KAPA Library Quantification kit (KAPA Biosystems, CapeTown, South Africa) and Tapestation (Agilent, USA) for molarity and fragment length estimation, respectively. Sequencing of the PCR-free library was performed on an Illumina NovaSeq 6000 sequencing platform using the run configuration of 2 × 150 bp.

### 2.3 Read pre-processing

A total of 128 Gbp of sequence reads was generated from sequencing. Reads were pre-processed with the removal of poly-Gs at the 3′ ends (*–poly_g_*min*_len 1*) [5] followed by adapter- and quality-trimming with Trimmomatic [6] (*ILLUMINACLIP:2:30:10, AVGQUAL:20, MINLEN:75*). This resulted in a clean set of 125.5 Gbp in approximately 838.5 million reads.

### 2.4 Whole genome assembly and annotation

The haploid genome size for *Clarias macrocephalus* was first estimated using a k-mer based approach. Pre-processed reads of mitochondrial origin were removed through the alignment of reads to the mitochondrial genome (assembly described in Section 2.6, accession number: MT109097) with bowtie2 v2.3.3.1 (-*I 100 -X 500* and using the *–un-conc* parameter for pairs of reads that did not align concordantly). Jellyfish was used to obtain a frequency distribution of k-mers (*k* = 19, 21 and 25). These k-mer distributions were processed with GenomeScope [7] (max *kmer coverage* disabled), which estimated an average haploid genome size of 881 Mbp, a 0.5% heterozygosity level and 70.7 to 81% of unique genome content (Supplementary Data 1).

MaSuRCA [8] was used to assemble the sequence reads in a *de novo* manner. The resulting assembly was subsequently run through Purge Haplotigs pipeline [9] to eliminate artefactual scaffolds caused by possible haplotigs. This produced a final assembly of 883 Mbp, a size that is consistent with the expected genome size for this species. The assembly is presented in 27,833 scaffolds with a scaffold N_50_ length of 80.8 kbp and contains 96.3% and 87.6% of complete BUSCOs (v3.0.2) based on a set of 978 metazoan and 4584 Actinopterygii orthologs, respectively (Table 1) (Supplementary Data 2), which exceeds other recently sequenced catfish genome assemblies [4].

To obtain the repeat profile of the bighead catfish genome, RepeatModeler was used to build a *de novo* repeat library, the output of which was used by RepeatMasker to mask repetitive regions in the assembly. This process masked 38.28% of the assembly and identified the DNA/TcMar-Tc1 transposon as the most abundant repeat type (Supplementary Data 3). This is consistent with the observation of elevated levels of this transposon in the *Ictalurus punctatus* genome [10] and freshwater fish in general [10,11].

The MAKER pipeline [12] was used to predict gene boundaries and coding sequences within the assembly. For this purpose, two types of sequence information were provided to the pipeline to increase the accuracy and sensitivity of predictions. Firstly, publicly-available *C. macrocephalus* RNA reads were downloaded from NCBI's SRA database (accession: SRR3161876), pre-processed with Trimmomatic and assembled with Trinity v2.8.5 (*–SS_lib_type FR*) [13]. This produced a set of 124,748 transcripts. Secondly, all protein sequences from SwissProt (accessed on January 31st, 2020) was provided as protein homology evidence. Both sets of information were used by MAKER to produce initial gene models in its first iteration (*est2genome = 1, protein2genome = 1*). These gene models were then used to train two *ab initio* gene predictors used in a second iteration of prediction. MAKER was run for a third iteration with gene models retrained with the output from the previous iteration. This process predicted 21,124 gene sequences with Annotation Edit Distances (AED) of ≤ 0.5 (Supplementary Data 4). A small AED indicates agreement of the annotation to aligned evidences while a AED=1 shows no evidence support for the annotation [14]. At least 98% of these protein sequences possess a functional domain (98% from InterProScan analysis [15]) or show homology (evalue 1e^−10^, 98.4% from BLASTP analysis [16]) to an existing sequence on NCBI's non-redundant database.

### 2.5 Enriched genes in *Clarias macrocephalus*

A distinctive feature of the genome of the related air-breathing walking catfish *Clarias batrachus* was an expansion of myoglobin (*mb*), olfactory receptor related to class A G protein-coupled receptor 1 (*ora1*) and sulfotransferase 6b1 (*sult6b1*) genes compared to non-air-breathing fishes [3]. We examined the generality of this finding by downloading the protein sequences of 17 other fish species, mostly from the Otophysi clade (https://www.ncbi.nlm.nih.gov/Taxonomy/Browser/wwwtax.cgi?id=186626), from Ensembl. A list of species and Ensembl identifier is available in Table 2 which excludes protein sequences for *C. batrachu*s which were not made publicly available by the authors [3] and therefore could not be included in this analysis. Groups of orthologous proteins were identified by OrthoFinder v2.3.3 [17], which uses DIAMOND *blastp* (*–more-sensitive, -e 0.001*) for all pairwise alignments, resulting in the identification of orthogroups for *mb, ora1* and *sult6b1* genes. Sequences for each gene were aligned with MAFFT, trimmed with Gblocks (allowing for gaps) and finally used by IQ-TREE [18] to build Maximum-likelihood phylogenetic trees. Orthogroups and phylogenetic trees are available in Supplementary Data 5. This analysis recovered substantially higher numbers of *mb* (14 copies) and *ora1* (10 copies) genes in the *C. macrocephalus* genome, clustered together in a phylogenetic clade (Fig. 2), relative to the genomes of other fish species (mostly 1 or 2 copies). These results are similar to the finding for C. *batrachus* [3], however an expansion was not observed for the *sult6b1* gene, which was recovered in copy numbers similar to that in other fish genomes such as the channel catfish (*Ictalurus punctatus*) and the stickleback (*Gasterosteus aculeatus*) (Table 2). The *mb, ora1* and *sult6b1* genes were found on 12, 3 and 4 scaffolds, respectively, with some evidence of tandem duplications similar to that reported for *C. batrachus* [3].

**Table 2:** Number of gene copies for *mb, ora1* and *sult6b1* in the genomes of *Clarias macrocephalus* and other fishes.

Species name	Ensembl code	*mb*	*ora1*	*sult6b1*
*Astyanax mexicanus*	ENSAMXP	1	1	2
***Clarias macrocephalus***	**N/A**	**14**	**10**	**5**
*Cyprinus carpio*	ENSCCRP	2	1	1
*Danio rerio*	ENSDARP	5	1	1
*Electrophorus electricus*	ENSEEEP	1	3	5
*Gadus morhua*	ENSGMOP	1	1	4
*Gasterosteus aculeatus*	ENSGACP	2	1	6
*Ictalurus punctatus*	ENSIPUP	1	1	7
*Oreochromis niloticus*	ENSONIP	1	1	2
*Oryzias latipes*	ENSORLP	1	1	3
*Poecilia formosa*	ENSPFOP	1	1	1
*Poecilia latipinna*	ENSPLAP	1	1	3
*Pygocentrus nattereri*	ENSPNAP	1	1	2
*Sinocyclocheilus anshuiensis*	ENSSANP	1	1	3
*Sinocyclocheilus grahami*	ENSSGRP	1	1	2
*Sinocyclocheilus rhinocerous*	ENSSRHP	2	1	5
*Takifugu rubripes*	ENSTRUP	2	1	1
*Tetraodon nigroviridis*	ENSTNIP	5	3	1

### 2.6 Mitochondrial genome assembly and annotation

A subset of 20 million pre-processed reads were assembled with the IDBA-UD assembler [19], which is used for the assembly of datasets with uneven read depths, to extract the *C. macrocephalus* mitochondrial genome using the genome skimming approach [20], recovering a circular mitochondrial genome of 16,511 bp. This was annotated with MITOS, followed by manual adjustments of gene boundaries based on homology to protein sequences in NCBI's non-redundant database, generating a complete repertoire of 37 genes typically found in an animal mitochondrial genome. Fig. 2 shows the arrangement of 13 protein-coding genes, 22 transfer RNAs and 2 ribosomal RNAs visualized with OrganellarGenomeDRAW. The mitochondrial COI gene has 100% identity sequences for the species on NCBI and the whole mitogenome has the highest similarity with other *Clarias* species on NCBI (91.6–89.4%). The mitochondrial genome is available on NCBI under the accession number MT109097.

## Declaration of Competing Interest

The authors declare that they have no known competing financial interests or personal relationships which have, or could be perceived to have, influenced the work reported in this article.

## Appendix Supplementary materials

Image, application 1
